# New combinations for Pacific endemic species: Marquesan Poaceae, and Micronesian Myrtaceae

**DOI:** 10.3897/phytokeys.28.6139

**Published:** 2013-11-04

**Authors:** Michael W. Tornabene, Warren L. Wagner

**Affiliations:** 1 Department of Botany, MRC-166, Smithsonian Institution, P.O. Box 37012, Washington DC 20013-7012

**Keywords:** Marquesas Islands, Micronesia, Pacific, *Cenchrus*, *Syzygium*, *Pennisetum*, *Eugenia*, Myrtaceae, Poaceae

## Abstract

As part of the preparation for a comprehensive online flora of Pacific oceanic islands, numerous taxonomic changes have been necessary, primarily due to a new wealth of global molecular phylogenetic studies on genera that include Pacific islands species. In order to compile an accurate checklist of the Pacific island flora with up-to-date taxonomies, we are moving several species to their currently accepted genera. Two Marquesan *Pennisetum* Rich. are transferred to *Cenchrus* L. with the new combinations *Cenchrus articularis* (Trin.) M. Tornabene & W.L. Wagner, and *Cenchrus henryanus* (F. Br.) M. Tornabene & W.L. Wagner. A key to Marquesas *Cenchrus* is also provided to differentiate the two species. Additionally, one species of *Eugenia* L. is transferred to *Syzygium* Gaertn. with the new combination *Syzygium stelechanthoides* (Kaneh.) M. Tornabene & W.L. Wagner in accord with the aforementioned studies.

## Systematics

### Marquesas Islands *Cenchrus*

A recent comprehensive molecular study of the genera *Cenchrus*, *Pennisetum*, and *Odontelytrum* by Chemisquy et al. (2010), has helped resolve a troublesome group in Poaceae. Their results showed that *Cenchrus*, *Pennisetum*, and *Odontelytrum* form a single monophyletic clade, with *Cenchrus* and *Odontelytrum* nested inside *Pennisetum* suggesting that the three genera should be unified into one inclusive genus, thereby transferring all species into *Cenchrus* as it is the genus with nomenclatural priority. Chemisquy et al. (2010) provided the needed combinations in their paper, but missed the species from the Marquesas Islands. Recent work on the taxonomy of the Marquesas populations for the online flora of the Marquesas suggest that there are two species described in the genus *Pennisetum* (Wagner and Lorence 2002, 2011). We, in accord with the work of Chemisquy et al., are transferring these two endemic species to *Cenchrus*. A short key prepared by Robert Soreng (US) has been included to delineate the two Marquesan species of *Cenchrus*.

#### 1. Cenchrus articularis

(Trin.) M. Tornabene & W.L. Wagner comb. nov.

urn:lsid:ipni.org:names:77133592-1

http://species-id.net/wiki/Cenchrus_articularis

Fig. 1

Pennisetum articulare (Basionym) Trin., Spreng. Neue Entdek. 2: 77. 1821. Type. MARQUESAS ISLANDS: Nuku Hiva. Probably Mar. 1818, [M. Wormskiold s.n.] (Holotype: LE).
Pennisetem identicum Steud. ex Jard.,  Mem. Soc. Sci. Nat. Cherbourg 5: 325. 1857. Type. MARQUESAS ISLANDS. Nuku Hiva, 1853?, D.E.S.A. Jardin 134 (Holotype: NTM?; Isotype: P[2]!).
Pennisetum simeonis F. Br., Bernice P. Bishop Mus. Bull. 84: 61. 1931. Type. MARQUESAS ISLANDS. Nuku Hiva. Coastal cliff and slopes, 10-800 m, 1922, S. Delmas s.n. (Lectotype: BISH- 92758!, designated by St. John, 1976, 418). Brown did not designate a type for this species in which he described three varieties. St. John selected the first of the three (var. intermedium) which becomes the autonym. *Pennisetum simeonis* var. *intermedium* F. Br., Bernice P. Bishop Mus. Bull. 84: 62. 1931.
Pennisetum simeonis var. *pedicellatum* F. Br., Bernice P. Bishop Mus. Bull. 84: 62. 1931. Type. MARQUESAS ISLANDS. Ua Huka: Open slopes and coastal cliffs, 15-1500 m, 24 Apr. 1921, F.B.H. Brown & E.D.W. Brown 360 (Holotype: BISH- 92750!).
Pennisetum simeonis var. *purpureum* F. Br., Bernice P. Bishop Mus. Bull. 84: 63. 1931. Type. MARQUESAS ISLANDS. Hiva Oa: Coastal cliff, dry slopes. 6–760 m, s.d., P.S. Delmas 12a (Holotype: BISH- 92751!; Isotype: US!).

##### Distribution.

Marquesas Islands, common on shore cliffs on the islands of Hatutaa, Nuku Hiva, Ua Huka, Hiva Oa, and Fatu Hiva, 0–900 m.

##### Note.

During research on the type of *Pennisetum articulare* Trin., an important discrepancy became apparent. The specimen has always been assumed to have been collected by Kyber who was on numerous expeditions with Ferdinand von Wrangel between 1821 and 1827 (Wrangel and Sabine 1840; Soreng et al. 1995). This would initially appear to be supported by the information contained on the Holotype label, “Exped. Sub Navarrho F.P. de Wrangel” and a small note in illegible German script labeled Kyber, 1827. However, the species was published in 1821, at least two years prior to when Kyber was reported to have arrived in the Pacific with Von Wrangel., (Wrangel and Sabine 1840).

After an extensive review of the relevant literature, which revealed that Ferdinand von Wrangel was in the Marquesas Islands in mid-March 1818 with the Danish botanist Marten Wormskjold (Golvonin 1979), we have concluded that Marten Wormskjold likely collected the specimens during this time, leaving his collections with Von Wrangel to be delivered to Trinius for identification. This theory is substantiated by the fact the all of the writing on the original label is Trinius’ handwriting, except for Kyber’s note. Subsequently the specimen was believed to have been returned to Von Wrangel providing the opportunity in 1827 for Dr. Kyber, the previously presumed collector, to examine the specimen and make additional notes.

**Figure 1.**
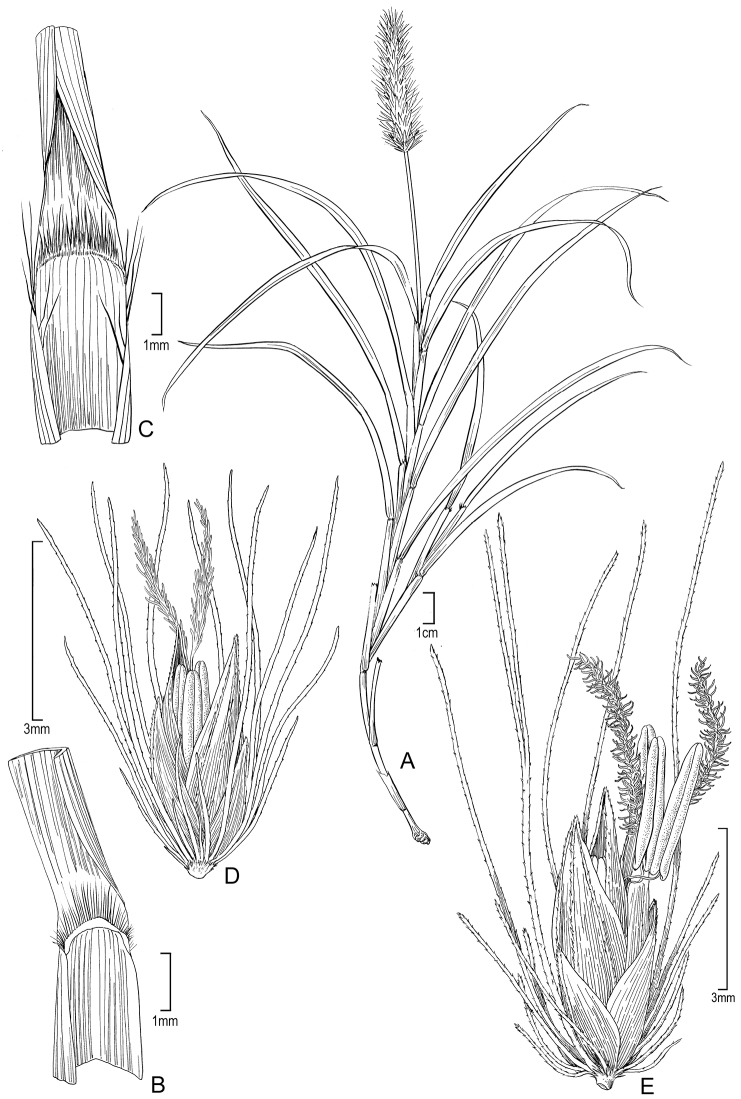
*Cenchrus articularis* (Trin.) M. Tornabene & W.L. Wagner **A** Habit, Perlman 10069 (US) **B** Ligule, Wood 10133 (US) **C** Ligule, Florence 9421 (US) **D** Spikelet with one floret, Wood 10112 (US) **E** Spikelet with two florets, Wood 10112 (US). Illustration by Alice Tangerini.

#### 2. Cenchrus henryanus

(F. Br.) M. Tornabene & W.L. Wagner comb. nov.

urn:lsid:ipni.org:names:77133590-1

http://species-id.net/wiki/Cenchrus_henryanus

Fig. 2

Pennisetum henryanum (Basionym) F. Br., Bernice P. Bishop Mus. Bull. 84: 61. 1931. TYPE. MARQUESAS ISLANDS: Nuku Hiva: Hakaui, precipitous slope of cliff, 0-100 m, 16 Jun 1921, F.B.H. Brown & E.D.W. Brown 454 (Holotype: BISH- 188906!).
Pennisetum henryanum F. Br. var. *longisetum* F. Br., Bernice P. Bishop Mus. Bull. 84: 61. 1931. Type. MARQUESAS ISLANDS. Nuku Hiva: near Hakaui, Tovii region, 1000 m, 2 Jul 1921, F.B.H Brown & E.D.W. Brown 471 (Holotype: BISH- 92752!)
Pennisetum henryanum F. Br. var. *pluristylum* F. Br., Bernice P. Bishop Mus. Bull. 84: 61. 1931. Type. MARQUESAS ISLANDS. Nuku Hiva: Taiohae, precipitous slopes of sea cliff, 20-800 m, 14 Jun 1921, F.B.H Brown & E.D.W. Brown 454A (Holotype: BISH- 92793!)

##### Distribution.

Endemic to Marquesas Islands on Nuku Hiva and Fatu Hiva, from 800–1130 m. *Cenchrus henryanus* is known from only a single collection from Fatu Hiva.

**Figure 2.**
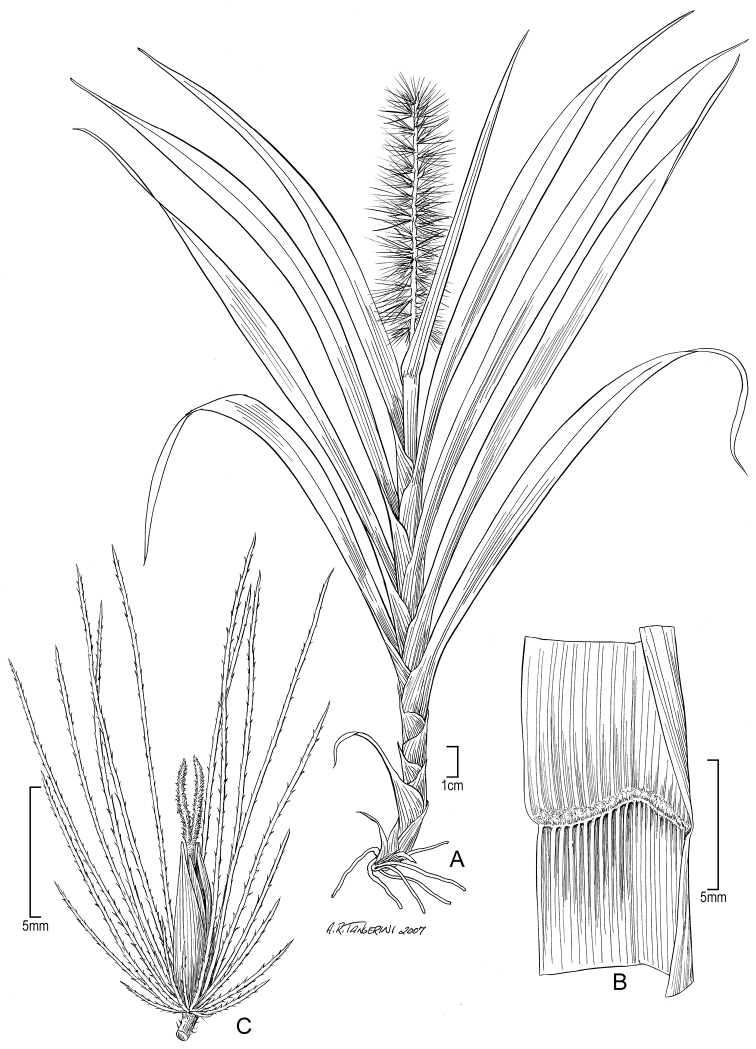
*Cenchrus henryanus* (F. Br.) M. Tornabene & W.L. Wagner **A** Habit **B** Ligule **C** spikelet. Drawn from Perlman 10106 (US). Illustration by Alice Tangerini.

#### Key to Native Species of *Cenchrus* in Marquesas Islands

**Table:** 

1	Longest Ligule hairs 1 (–1.5) mm; leaf-blades slender (but up 1.2 cm wide at the broadest point which is well above the base); longest bristles below the spikelets less than 1–1.3 cm long; upper mid-culm internodes often ca. 3 cm long	*Cenchrus articulare*
2	Ligule hairs 0–0.5 mm; leaf-blades usually 1 cm wide (at least at the base of the leaf); longer bristles below the spikelets (1.5–) 1.7—2 cm long; upper mid-culm internodes contracted, mostly 2–2.5 (–3) cm long	*Cenchrus henryanus*

### Micronesian *Syzygium*

According to multiple recent molecular studies examining the relationships between species in one of the major lineages within the Myrtaceae, the Syzygium group, one species of endemic Micronesian *Eugenia* L. should be placed in *Syzygium* P. Browne (Craven and Biffin 2010; Biffin et al. 2006). This confirms conclusions reached by Schmid (1972) based on morphological and anatomical data. We are making the necessary new combination here to make it available on the online checklist of Micronesia (Wagner et al. 2012).

#### 1. Syzygium stelechanthoides

(Kaneh.) M. Tornabene & W.L. Wagner comb. nov.

urn:lsid:ipni.org:names:77133591-1

http://species-id.net/wiki/Syzygium_stelechanthoides

Eugenia stelechanthoides (Basionym) Kaneh. Bot. Mag. (Tokyo). 46: 669. 1932. *Jambosa stelechanthoides* (Kaneh.) Hosok. J. Jap. Bot. 16: 544. 1940. Type. CAROLINE ISLANDS: Kosrae: In a Horsfieldia forest at the middle altitudes on Mt. Matante, 29 Jul 1933, T. Hosokawa 6219 (Holotype: TAI).

##### Distribution.

Caroline Islands, known only from the island of Kosrae.

##### Note.

In the recently published World Checklist of Myrtaceae, *Eugenia stelechanthoides* Kaneh. was placed into synonymy with *Syzygium stelechantha* (Diels) Glassman. In his original publication, Kanehira acknowledges the close relationship of the two species, but also notes two major differences that correlate with distribution. Specimens from Kosrae (*Syzygium stelechanthoides*) have much larger leaves, as well as winged branchlets, while the specimens from Pohnpei (*Syzygium stelechantha*) do not. Upon examination of specimens of both species from Pohnpei, and Kosrae, as well as additional islands in the Caroline archipelago at US, only specimens from Kosrae exhibited the characteristics of *Syzygium stelechanthoides* described by Kanehira. Therefore, until a more in depth study can be performed on the Syzygium of the area, we find it best to maintain *Syzygium stelechanthoides* as a separate species.

## Supplementary Material

XML Treatment for Cenchrus articularis

XML Treatment for Cenchrus henryanus

XML Treatment for Syzygium stelechanthoides
